# Fibrin clot quality in acutely ill cirrhosis patients: Relation with outcome and improvement with coagulation factor concentrates

**DOI:** 10.1111/liv.15132

**Published:** 2021-12-20

**Authors:** Annabel Blasi, Vishal C. Patel, Eva N. H. E. Spanke, Jelle Adelmeijer, Marilena Stamouli, Ane Zamalloa, Eleanor Corcoran, Andrea Calvo, Javier Fernandez, William Bernal, Ton Lisman

**Affiliations:** ^1^ Anesthesiology Department Hospital Clínic and University of Barcelona Barcelona Spain; ^2^ Institute d'Investigacions Biomèdica Agustí Pi i Sunyer (IDIBAPS) Barcelona Spain; ^3^ Institute of Liver Studies & Transplantation King's College Hospital NHS Foundation Trust London UK; ^4^ Liver Sciences School of Immunology & Microbial Sciences King's College London UK; ^5^ Institute of Hepatology Foundation for Liver Research London UK; ^6^ Surgical Research Laboratory Department of Surgery University Medical Center Groningen University of Groningen Groningen The Netherlands; ^7^ Department of Critical Care King's College Hospital NHS Foundation Trust London UK; ^8^ Liver Unit Institut de Malalties Digestives i Metabòliques Hospital Clínic and University of Barcelona Barcelona Spain; ^9^ Section of Hepatobiliary Surgery and Liver Transplantation Department of Surgery University Medical Center Groningen University of Groningen Groningen The Netherlands

**Keywords:** blood coagulation, cirrhosis, factor XIII, fibrin, haemorrhage

## Abstract

**Background & Aims:**

Patients with liver disease may acquire substantial changes in their hemostatic system, which are most pronounced in patients who are critically ill. Changes in the quality of the fibrin clot in critically ill patients have not been studied in detail. Here we assessed markers of fibrin clot quality and effects of coagulation factor concentrates in patients with acutely decompensated (AD) cirrhosis and acute on chronic liver failure (ACLF).

**Methods:**

We measured plasma levels of fibrinogen, factor XIII, prothrombin and performed thrombin generation assays in 52 AD patients, 58 ACLF patients and 40 controls. In addition, we examined the effects of coagulation factor concentrates on functional assays of fibrin quality.

**Results:**

We found increased thrombin generating capacity in both AD and ACLF in comparison with healthy controls. Plasma levels of prothrombin, fibrinogen, and factor XIII were lower in patients compared to controls, appeared lower in ACLF compared to AD patients, and were related to clinical outcomes. Fibrinogen concentrate, but not factor XIII or prothrombin complex concentrate, improved clot quality in vitro. Prothrombin complex concentrate increased the resistance of the clot to break down.

**Conclusions:**

We have demonstrated elevated thrombin generation but decreased plasma levels of prothrombin, fibrinogen and FXIII in acutely ill patients with cirrhosis. In addition, we showed that fibrinogen concentrate and PCCs, but not factor XIII concentrate, improve clot properties in patient plasma. Whether there is true clinical benefit from coagulation factor concentrates in prevention or treatment of bleeding requires further study.

**Lay summary:**

Patients with liver diseases are at risk of bleeding, but mechanisms involved in this bleeding risk are incompletely understood. We studied components that determine the stability of the blood clot and found that concentrations of certain proteins involved in clot stability are present in low levels in acutely ill patients with liver disease. We furthermore demonstrated that some clinically available drugs improve the stability of blood clots from these patients in a test tube.

**Abbreviations**
ACLF: acute‐on‐chronic liver failure
AD: acutely decompensated
CLT: clot lysis time
ETP: endogenous thrombin potential
Ks: Darcy's constant
MELD: model of end‐stage liver disease
PCC: prothrombin complex concentrate
PT/INR: prothrombin time/international normalized ratio
SOFA: sequential organ failure assessment
TBS: tris‐buffered saline
tPA: tissue‐type plasminogen activator

## 1 INTRODUCTION

Liver diseases are frequently associated with profound hemostatic changes. As these changes concern both pro‐ and antihemostatic pathways, the net effect is a rebalanced hemostatic system.^1^, ^2^ This hemostatic rebalance appears even maintained in the sickest patients, including those with acutely decompensated (AD) cirrhosis and acute‐on‐chronic liver failure (ACLF).^3^ However, the hemostatic balance in patients with advanced liver disease is much less stable compared to individuals with adequate liver function, and perturbation of the hemostatic balance leads to both bleeding and thrombosis in these fragile patients.^4^

Laboratory and clinical studies have led to a better understanding of hemostatic malfunction and its treatment in patients with liver disease. Although well‐designed clinical studies are still lacking, recent clinical guidance documents advise limited use of prophylactic administration of blood products such as fresh frozen plasma,^5^, ^6^ which has very little prohemostatic activity in patients with liver disease.^7^, ^8^, ^9^, ^10^ Factor concentrates may be much more useful to prevent or treat bleeding as they have increased prohemostatic activity and lack issues that complicate the use of blood products such as fluid overload and risk of other transfusion‐associated complications. Such factor concentrates include prothrombin complex concentrates (PCCs), fibrinogen concentrate, and factor XIII concentrate. Administration of these concentrates may be guided by laboratory tests including the PT/INR (for PCCs), thromboelastography (for all concentrates), and fibrinogen assays (for fibrinogen concentrate).

Currently, factor concentrates are used in some centres in patients with liver disease,^11^, ^12^, ^13^, ^14^, ^15^ but without a clear evidence‐base. Use of concentrates is in part driven by studies showing an increased bleeding risk in those patients with factor deficiencies (including fibrinogen^15^, ^16^ and factor XIII^17^), or a further decrease in factor levels with specific external triggers (such as acute kidney injury which has been linked to a specific exaggeration of FXIII deficiency in cirrhosis^18^, ^19^). However, it has not been established whether normalization of factor levels reduces bleeding risk, and no causal connection between factor levels and bleeding risk has been demonstrated. A recent retrospective study of critically ill patients with cirrhosis showed no independent association of low fibrinogen levels with mortality or bleeding events and concluded that low fibrinogen levels were a reflection of disease severity rather than being causally related to bleeding.^15^ That study also showed no reduction in bleeding with cryoprecipitate transfusion administered either prophylactically or in actively bleeding patients. However, it is largely unknown whether factor concentrates lead to functional improvement of the fibrin clot, as clinically used laboratory tests do not take important physiological aspects of clot stability into account. The fibrin clot requires resistance to the fibrinolytic system, which is incompletely assessed using viscoelastic tests that are insensitive for a hypofibrinolytic state,^20^ and may not be sensitive for mild hyperfibrinolysis.^21^ In addition, the permeability of the clot, which is a function of clot pore size, is related to bleeding and thrombotic disease, but is not assessed in any clinically used tests.^22^ Finally, factor XIII‐dependent red blood cell retention within contracting clots has recently been identified as an important factor in clot stability.^23^

Here, we assessed thrombin generating capacity, fibrinogen levels and factor XIII levels in relation to disease severity and outcome in patients with AD and ACLF. As we found markers of fibrin clot quality (fibrinogen and factor XIII levels), but not thrombin generating capacity to be associated with disease stage, severity and outcome, we subsequently assessed effects of in vitro addition of clinically available factor concentrates on functional markers of clot stability.

## 2 MATERIALS AND METHODS

### 2.1 Patients

Between February 2018 and September 2018, adult patients with acutely decompensated cirrhosis (AD, n = 52) or acute on chronic liver failure (ACLF, n = 58) consecutively admitted to King's College Hospital London (United Kingdom) and Hospital Clinic Barcelona (Spain) who gave written informed consent were included in this study. The NRES Committee London‐Westminster (12/LO/1417) and the Medical Ethical Committee Hospital Clinic Barcelona (2017/0948) approved the study protocol which was in accordance with the Helsinki Declaration of 1975. Informed consent or assent was obtained from participants or their personal consultees. Exclusion criteria for this study were acute liver failure, known congenital coagulation disorders, the use of anticoagulants or platelet function inhibitors, pregnancy, Human Immunodeficiency Virus positivity, or evidence of disseminated malignancy. Cirrhosis was defined by the presence of 2 or more of: (a) histological evidence of cirrhosis on liver biopsy, (b) laboratory abnormalities consistent with cirrhosis or (c) radiological findings consistent with cirrhosis and portal hypertension. Acute decompensation of cirrhosis and ACLF were defined and graded according to number of organ failures in concordance with criteria reported in the CANONIC study.^24^ Patients were followed‐up until 30 days after discharge, death or liver transplantation, whatever happened first. Healthy controls aged >18 years (n = 40) were enrolled to establish reference values for the various laboratory tests performed and excluded those with a personal history of thrombotic or liver disease, chronic medical conditions, or current use of anticoagulants, platelet function inhibitors, or oral contraceptives.

### 2.2 Data collection

We collected baseline data on patient demographics, co‐morbidities, biochemistry and disease severity scores. The severity of liver disease was evaluated with sequential organ failure assessment (SOFA), CLIF‐AD, CLIF‐ACLF, model of end‐stage liver disease (MELD), and Child‐Pugh scores. Sepsis syndrome was defined according to the SEPSIS‐3 guidelines.^25^ Data on haemorrhagic or thrombotic events and on transfusion requirements were collected throughout hospitalization. Bleeding events were defined according to the following criteria: fatal bleeding, symptomatic bleeding in a critical area or organ and/or bleeding causing a fall in haemoglobin level of ≥2 g/L or leading to transfusion ≥2 units of packed red cells.

### 2.3 Blood samples

Blood samples were collected in sodium citrate‐containing vacutainer tubes (0.129 M) from an arterial line, central venous catheter, or by standard peripheral venous phlebotomy within the first 2 days of admission or after the development of ACLF. Samples were obtained prior to the administration of blood products, anticoagulants, or platelet function inhibitors. Within 2 hours after the blood draw, the sample was centrifuged at 2000 and 10 000 g respectively for 10 minutes at ambient temperature. Plasma was stored at −80°C until it was used for analyses.

### 2.4 Endogenous thrombin potential

We measured thrombomodulin‐modified thrombin generation using Calibrated Automated Thrombography using protocol and reagents from Thrombinoscope BV, Maastricht, The Netherlands.

### 2.5 Plasma protein levels

Prothrombin and fibrinogen levels were measured on an automated coagulation analyser (ACL 300 TOP) with reagents and protocols from the manufacturer (Werfen). Factor XIII activity levels were measured as described previously.^26^

### 2.6 Clot lysis assay

Lysis of a TF‐induced clot by exogenous tissue plasminogen activator (tPA) was studied by monitoring changes in turbidity during clot formation and subsequent lysis as described previously.^27^ Clot lysis time was determined as the time from the midpoint of the clear to maximum turbid transition, which characterizes clot formation, to the midpoint of the maximum turbid to clear transition, which represents clot lysis.

### 2.7 Permeability assays

Plasma clotting mixtures for permeation measurements were prepared as described.^28^ Immediately after mixing, 100 μL of the clotting mixture was carefully transferred to a 4.5‐cm plastic tip with a roughened interior surface, which was cut off from a 1‐mL Costar pipette tip. The clot mixture was left to consolidate in a humidified chamber at room temperature for 2 h. The plastic tip was then connected through a flexible silicon tube to a syringe containing tris‐buffered saline (TBS) with a 4‐cm pressure drop. Upon connection, TBS was left to permeate through the fibrin clot network for 1.5 h to wash any other, non‐fibrin, plasma components, such as albumin. Then, measurements were performed in duplicate by collecting drops passing through the clot in a pre‐weighed Eppendorf tube and weighing the total volume of liquid in the tube every 30 min for 2 h. These measurements were averaged. Clot permeability was ultimately determined by calculating Darcy's constant (Ks), which is a measure of the pore size of the fibrin network through which liquid may pass, as previously described.^29^

### 2.8 Red cell retention assays

Clot retraction with subsequent red cell extrusion from the clots was performed as described.^30^ Plasma was mixed with isolated platelets and red blood cells from healthy blood donors with blood group 0. This reconstituted blood was clotted with tissue factor (Innovin Siemens Healthcare, 1:12:000 final dilution) and Calcium chloride (10 mM final concentration) in siliconized wells for 2 hours at 37°C. Clots were weighed after removing adherent liquid, and the haemoglobin level in the supernatant (diluted with phosphate‐buffered saline) was estimated by absorbance measurements at 575 nm. The percentage of extruded RBCs was calculated by comparing the absorbance of reconstituted blood and the supernatant after clot formation and retraction.

### 2.9 Addition of factor concentrates

We performed clot lysis assays, permeability assays, and red cell retention assays after addition of vehicle, fibrinogen concentrate (Haemocomplettan P; CSL Behring, 1 g/L final concentration), FXIII concentrate (Cluvot; CSL Behring, 15 μg/ml final concentration), or prothrombin complex concentrate (Cofact; Sanquin, 0.5 IU/ml final concentration) to the plasma samples prior to the analysis or prior to mixing with platelets and red blood cells. Clot lysis assays were performed with samples from all patients and controls, and permeation assays were performed with plasma from 8 randomly chosen healthy controls and 12 randomly selected patients (6 AD, 6 ACLF), as permeation assays are very labour‐intensive. For red cell retention assays, we used pooled plasma of 10 randomly selected patients with AD, 10 patients with ACLF, and 10 healthy individuals, as insufficient plasma sample was available to perform assays with plasma from individual patients.

### 2.10 Statistical analyses

Statistical analyses were performed using SPSS statistics, version 23 (IBM Inc.) and GraphPad Prism. Data were presented as means with standard deviation or medians and interquartile ranges for continuous variables as appropriate, and as percentages for categorical variables. Means of two groups were compared by Student's *t* test or Mann‐Whitney *U* test as appropriate. Multiple groups were compared using One‐way ANOVA (with the Bonferroni posttest) or Kruskal‐Wallis *H* test (with Dunn's posttest) as appropriate. Pearson's correlation coefficient was used to assess the correlation between variables. A *P*‐value <0.05 was considered significant.

## 3 RESULTS

### 3.1 Patient characteristics

We have studied endogenous thrombin potential and plasma levels of prothrombin, fibrinogen and factor XIII in 52 adult patients with AD cirrhosis and 57 adult patients with ACLF and compared these levels with plasma concentrations in 40 healthy controls. Patient demographics, clinical and laboratory data are presented in Table 1. Healthy controls were younger than patients (35 [28‐43] years and 45% were male). Among patients with ACLF, 27 (47%) had ACLF‐1, 13 (23%) ACLF‐2, and 17 (30%) ACLF‐3, at inclusion.

**TABLE 1:** Demographic and laboratory data of the study population

	AD n = 52	ACLF n = 57	*P*
Age, years	58(50‐67)	59(49‐66)	ns
Aetiology			ns
Alcohol	32	33	
Viral	0	11	
NASH	10	6	
Biliary	3	2	
Other	7	5	
Male (n)	29	40	ns
SOFA score	4 (3‐6)	8 (6‐11)	<0.001
CLIF‐SOFA score	60 (47‐108)	87 (74‐97)	<0.001
MELD	15 (11‐21)	27 (23‐35)	<0.001
Child‐Pugh, points	9 (7‐10)	10 (8‐12)	0.001
Haemoglobin, g/dl	80 (86‐110)	85 (77‐99)	<0.001
Na, mmol/L	136 (132‐139)	135 (132‐141)	ns
Urea, mmol/L	5 (3‐8)	8 (4‐9)	0.007
Creatinine, µmol/L	73 (54‐109)	211 (134‐282)	<0.001
Bilirubin, µmol/L	39 (28‐101)	100 (40‐381)	0.001
Gamma glutamyl transpeptidase, IU/L	77 (43‐145)	70 (42‐1589)	ns
Alkaline phosphatase, IU/L	120 (82‐181)	116 (80‐142)	ns
Aspartate transaminase, IU/L	62 (44‐100)	65 (40‐108)	ns
Albumin, g/L	29 (26‐33)	29 (24‐33)	ns
Platelets × 10^9^	88 (62‐127)	70 (38‐107)	0.03
Fibrinogen, g/L	2.2 (1.4‐2.9)	1.8 (1.1‐2.5)	ns
INR	1.4 (1.3‐1.8)	1.7 (1.4‐2.6)	<0.001
APTT, seconds	36 (30‐43)	41 (33‐56)	0.005

Note

Abbreviations: APTT: activated partial thromboplastin time. Shown are medians and interquartile ranges; MELD: model of end‐stage liver disease;INR: international normalized ratio; NASH: Non‐alcoholic steatohepatitis;SOFA: sequential organ failure assessment.

### 3.2 Endogenous thrombin potential and plasma levels of prothrombin, fibrinogen and factor XIII in relation to disease severity and outcome

We found increased thrombin generating capacity in both AD and ACLF in comparison with healthy controls, as indicated by high ETP values in patients. Plasma levels of prothrombin, fibrinogen, and factor XIII were lower in patients compared to controls and appeared lower in ACLF compared to AD patients (Table 2). We then assessed these laboratory measures in patients stratified according to clinical scores and presence or absence of individual organ failure (Tables S1 and S2). ETP values were very similar between patients with high or low SOFA or CLIF scores which assess organ failure, and consequently were similar between patients with or without individual organ failures. ETP values were also similar between patients with and without active infection. In contrast, plasma levels of prothrombin, fibrinogen and FXIII were substantially lower in those patients with high compared to those with low SOFA scores, which appears predominantly determined by liver failure, coagulation failure, and hemodynamic failure. In contrast, prothrombin, fibrinogen and FXIII levels were similar in those with high or low CLIF scores, and in patients with and without infection.

**TABLE 2:** Endogenous thrombin potential and plasma levels of prothrombin, fibrinogen and factor XIII

	Controls (n = 40)	AD (n = 52)	ACLF (n = 57)
ETP (nM IIa * min)	451 (283‐604)	758 (668‐913) *P < .001	702 (528‐903) *P < .001
F II, %	96 (85‐102)	44 (30‐64) *P < .001	34(22‐44) *P < .001 ^≠^ *P* = .01
Fibrinogen, g/L	2.6 (2.4‐2.9)	2.1 (1.4‐2.9) *P = .01	1.8 (1.1‐2.5) *P < .001
F XIII, %	95 (85‐104)	44 (30‐59) *P < .001	32 (21‐43) *P < .001

Note

* vs controls, ^≠^ vs AD, Shown are medians and interquartile ranges.

In all patients combined, 35 (24%) experienced a bleeding event during hospitalization (8 patients in the AD group and 27 patients in the ACLF group), and more than half (63%) of these events were related to portal hypertension. Six thrombotic events were observed during hospitalization. Details of these events have been published previously.^31^ Baseline ETP and plasma levels of prothrombin, fibrinogen, and factor XIII were similar in patients who developed a thrombotic event during hospitalization. Prothrombin and factor XIII plasma levels were significantly lower in those patients who developed a bleeding episode during hospitalization but the differences between baseline prothrombin and FXIII levels between bleeders and non‐bleeders were small (Table 3). Indeed, the area under the receiving operating characteristic curve was 0.365 (0.257‐0.495, 95% confidence interval) for prothrombin and 0.376 (0.264‐0.502) for FXIII. Baseline prothrombin and fibrinogen levels were substantially lower in those patients who died within 30 days compared to those surviving beyond 30 days, with areas under the receiving operating characteristic curve of 0.726 (0.578‐0.873) for prothrombin and 0.846 (0.718‐0.974) for fibrinogen. In contrast, ETP values and plasma levels of FXIII were similar between survivors and non‐survivors (Table 4).

**TABLE 3:** Endogenous thrombin potential and plasma levels of prothrombin, fibrinogen and factor XIII in patients who experienced bleeding or thrombosis and those who did not during hospitalization

	Thrombosis yes (n = 6)/no (n = 103)	Bleeding yes (n = 35)/no (n = 74)
ETP, nM IIa * min	695 (581‐878)/750 (627‐911), *P* = ns	727 (640‐923)/750 (577‐889), *P* = ns
FII, %	37 (27‐48)/38 (25‐52), *P* = ns	34 (19‐43)/41 (28‐53), *P* = .02
Fibrinogen, g/L	2.1 (1.5‐2.5)/1.9 (1.2‐2.8), *P* = ns	1.7 (1.0‐2.5)/1.9 (1.4‐2.8), *P* = ns
FXIII, %	28 (13‐37)/35 (25‐51), *P* = ns	29 (18‐44)/35 (28‐56), *P* = .04

Note

Shown are medians and interquartile ranges.

**TABLE 4:** Endogenous thrombin potential and plasma levels of prothrombin, fibrinogen and factor XIII in patients who survived beyond 30 days of admission and those who did not

	Survived (n = 89)	Died (n = 17)	p
ETP, nM IIa * min	759 (629‐930)	641 (530‐836)	ns
F II, %	41 (29‐53)	20 (14‐29)	<0.001
Fibrinogen, g/L	2.1 (1.4‐2.8)	0.8 (0.6‐1.8)	<0.01
F XIII, %	35 (26‐50)	29 (18‐49)	ns

Note

Shown are medians and interquartile ranges.

### 3.3 In vitro *improvement of fibrin clot structure by clinically available factor concentrates*

In aggregate, thrombin generating capacity is elevated in patients and unrelated to clinical outcome, whereas plasma levels of prothrombin, fibrinogen and FXIII are decreased and related to disease severity or outcome. Normalization of plasma levels of these factors thus may offer clinical benefit, which in part may relate to improvements in fibrin clot structure, which is (among other factors) governed by prothrombin, fibrinogen, and FXIII levels. We therefore assessed the effects of clinically available factors concentrated on different aspects of clot structure, and compared effects of these concentrates between patients and controls. Plasma samples were spiked with clinically relevant doses of fibrinogen concentrate, factor XIII concentrate, and prothrombin complex concentrate.

#### 3.3.1 Resistance to fibrinolysis

Resistance to fibrinolysis was assessed using a plasma‐based assay in which a tissue factor‐induced clot is lysed by exogenous tissue‐type plasminogen activator. We only studied samples that had detectable clot lysis within the time frame of our experiment (9 patients with ACLF were excluded because they were profoundly hypofibrinolytic with CLTs >180 minutes).

**FIGURE 1:**
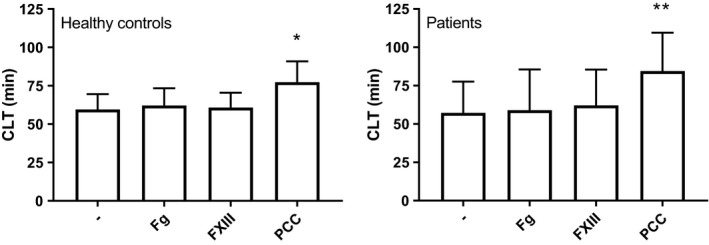
Effect of in vitro addition of factor concentrates on clot lysis time in healthy individuals and patients with AD and ACLF. Shown are means with standard deviations of 40 healthy controls and 94 patients. Nine patients with ACLF were not included in the analyses as the CLT without additions was already longer than 180 minutes (which is the maximum time this assay is run), from three other patients with ACLF insufficient plasma was available. *P < .05, **P < .01

Addition of fibrinogen or factor XIII had no effect on clot lysis times in healthy individuals or patients. In contrast, addition of prothrombin complex concentrate increased clot lysis times substantially in both controls and patients, and results were similar between patients with AD and ACLF (Figure 1). In line with a previous study from our group,^31^ CLT values were substantially higher in those patients with ACLF that had an active infection at the time of sampling (data not shown). Addition of PCCs increased CLT both in patients with and without an active infection. We have previously shown that PCCs substantially increase thrombin generation in patients and controls,^10^ and enhancement of thrombin generation leads to increased resistance to fibrinolysis due to enhanced activation of thrombin‐activatable fibrinolysis inhibitor (TAFI).^32^ Indeed, in a subgroup of 5 healthy controls and 13 patients we observed that the increase in clot lysis time was fully dependent on enhancement of TAFI activation as clot lysis times in the presence of a specific inhibitor of activated TAFI (carboxypeptidase inhibitor, 25 μg/ml) was identical in samples with and without PCC addition (data not shown). Although addition of factor XIII did not affect clot lysis times, even in individual patients with FXIII levels <20%, FXIII does contribute to resistance to lysis in this assay since complete inhibition of FXIIIa activity by T101 (final concentration 20 μM) shortened CLT in pooled normal plasma (From 64 ± 1 to 54 ± 1 minute, *P* < .001, n = 4).

#### 3.3.2 Plasma clot permeability

**FIGURE 2:**
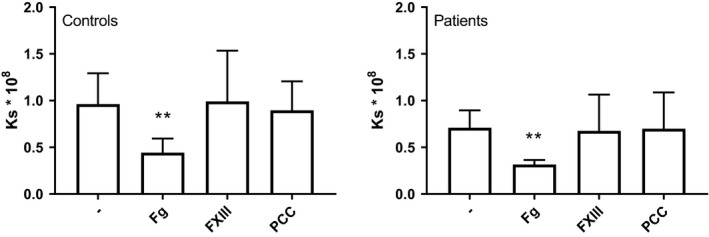
Effect of in vitro addition of factor concentrates on clot permeability expressed as Ks in healthy individuals and patients with AD and ACLF. Shown are medians with interquartile ranges of 8 healthy controls and 12 patients. **P < .01

Plasma clot permeability to liquid was determined as the second measure of clot quality. We added factor concentrates to plasma from 8 healthy individuals and 12 patients who had fibrinogen levels of 2.5 ± 0.6 g/L and 2.1 ± 1.5 g/L, respectively, and determined the average pore size of the fibrin clot (expressed as the Darcy constant, Ks). Addition of fibrinogen concentrate markedly reduced the Ks, whereas FXIII and PCC had no effect (Figure 2). Although addition of factor XIII did not affect Ks values, FXIII does contribute to clot stability in this assay since complete inhibition of FXIIIa activity by T101 (final concentration 20 μM) substantially lowered the Ks in pooled normal plasma (From 1.5 ± 0.3 to 0.7 ± 0.2, *P* < .01, n = 4).

#### 3.3.3 Red blood cell retention

**FIGURE 3:**
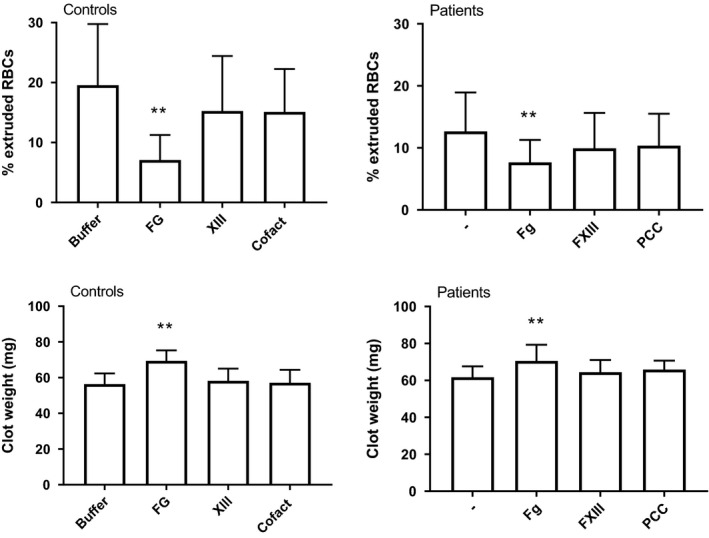
Effect of in vitro addition of factor concentrates on red cell extrusion and clot weight of full retracted clots from reconstituted blood of isolated platelets, red cells, and pooled plasma from 10 healthy individuals, 10 patients with AD, or 10 patients with ACLF. Shown are medians with interquartile ranges of 4 independent experiments. In patient graphs, 4 independent experiments with pooled AD plasma were combined with 4 independent experiments with pooled ACLF plasma. *P < .05, **P < .01

We mixed isolated platelets and red blood cells with pooled plasma from 10 healthy controls, 10 patients with AD, or 10 patients with ACLF, with fibrinogen concentrations of 2.6 ± 0.7 g/L, 2.3 ± 0.9, and 1.9 ± 1.4, respectively to which vehicle or factor concentrate was added. The reconstituted whole blood samples were clotted with tissue factor, and clots were allowed to retract for 2 hours. Retracted clots were weighed and the percentage of red cells extruded from the clots were determined by comparing haemoglobin levels in the freshly reconstituted blood and the supernatant after 2 hours of clot retraction. Each condition was analysed on four separate occasions. Addition of fibrinogen concentrate resulted in a decrease in RBC extrusion during clot contraction, and consequently in a higher weight of the contracted clots in both controls and patients. In contrast, addition of FXIII or PCCs only marginally reduced RBC extrusion and did not change clot weight (Figure 3). Although addition of factor XIII did not affect clot weight, FXIII does contribute to clot weight in this assay since complete inhibition of FXIIIa activity by T101 (final concentration 20 μM) lowered the clot weight in pooled normal plasma mixed with donor platelets and red cells (From 61 ± 2 to 48 ± 2 mg, *P* < .01, n = 4).

## 4 DISCUSSION

Here, we have confirmed and extended our previous data showing enhanced thrombin generating capacity with decreased plasma fibrinogen levels in patients with AD and ACLF.^3^ In addition, we show markedly decreased plasma levels of factor XIII in patients. Thrombin generating capacity did not relate to severity of disease, complications, or outcome. However, plasma levels of prothrombin, fibrinogen, and FXIII were markedly lower in sicker patients, with much lower levels in those who died within 30 days. Thus, important determinants of fibrin clot structure, but not thrombin generating capacity are related to severity of disease in patients with AD and ACLF. Although it is still unclear whether low levels of prothrombin, fibrinogen, and FXIII increase bleeding risk in patients with cirrhosis, we propose that structural defects in the fibrin clot may occur when levels become critically low. In this scenario, improvement of fibrin clot structure may be useful to prevent or treat bleeding in these difficult‐to‐treat patients. Using three distinct assays of fibrin clot structure, we show that fibrinogen concentrate and PCCs at clinically relevant doses lead to marked improvements in fibrin clot quality, whereas FXIII had no or very minor effects.

The observation that thrombin generation remains elevated even in the sickest patients with cirrhosis, and is unrelated to various indicators of disease severity, suggests defects in anticoagulant pathways compensate for defects in procoagulant pathways through the entire spectrum of cirrhosis severity.^33^ Plasma levels of individual coagulation proteins do worsen with the increasing severity of disease and may be predictors of outcome. We did, however, not confirm previous findings on an additional factor XIII decrease in patients with cirrhosis who develop acute kidney injury.^18^, ^19^ As clinical characteristics and the definition of kidney failure differed between studies, the role of factor XIII in this setting requires additional study.

It is unclear whether restoration of factor levels by infusion of factor concentrates reduces spontaneous or procedure‐related bleeding in patients with cirrhosis. A recent study found no clinical benefit from increasing fibrinogen plasma levels by cryoprecipitate.^15^ However, as we have recently demonstrated that the fibrin clot is unstable and particularly susceptible to break down by the fibrinolytic system in only a proportion of critically ill patients with cirrhosis,^31^ it may be that only a subset of patients would benefit from interventions improving resistance to fibrinolysis. We here demonstrate that fibrinogen concentrate and PCCs improve fibrin properties in vitro. Fibrinogen concentrate markedly decreased fibrin clot permeability and increased clot weight by increasing red blood cell retention, but had no effect on resistance to lysis. PCCs induced a marked increase in lysis resistance. Perhaps the combination of fibrinogen concentrate and PCC may benefit individual patients. Indeed, the combination of fibrinogen concentrate and PCC, guided by viscoelastic testing has been successfully used in hemostatic management during liver transplantation.^11^

Surprisingly, factor XIII concentrate had no effect on fibrin clot properties, even though factor XIII contributes to fibrin clot stability in all assays used as evidenced by the effect of full inhibition of factor XIIIa activity by T101. Apparently, only very small amounts of FXIII are sufficient for maximal clot protective effects in our assays, which aligns with the observation that only small amounts of factor XIII seem to prevent spontaneous bleeding in patients with congenital FXIII deficiency. Importantly, one previous study has shown low FXIII levels to be related to bleeding in patients with cirrhosis, and these authors suggested clinical benefits from FXIII infusion.^17^ Here, we also found lower FXIII levels in bleeders, although the difference with patients who did not bleed was small. Whether the association between low FXIII and bleeding is causal or merely indicates severity of disease is uncertain, and interventional clinical trials will be required to assess whether FXIII replacement may be beneficial in prevention or treatment of bleeding in patients with cirrhosis.

Previous in vitro studies have shown fibrinogen concentrate, PCCs, and FXIII to have beneficial effects on clot properties assessed by ROTEM in a model of trauma‐induced coagulopathy.^34^, ^35^ In this model, tranexamic acid also had beneficial effects on clot properties, and synergistic effects of tranexamic acid and coagulation concentrates were demonstrated. Why FXIII suppletion was effective in this model, but not in our experiment is not entirely clear, although the composition of the blood and the assays used were clearly different. Whether antifibrinolytic agents such as tranexamic acid or combinations of antifibrinolytics with coagulation factor concentrates are useful in critically ill patients with cirrhosis is uncertain. Importantly, the side effects observed in the HALT‐IT trial, a large randomized study of tranexamic acid use in gastrointestinal bleeding,^36^ which included a large number of patients with cirrhosis, may limit the use of this drug in patients with cirrhosis.^37^

In summary, we have demonstrated elevated thrombin generation but decreased plasma levels of prothrombin, fibrinogen and FXIII in acutely ill patients with cirrhosis. Plasma levels of these contributors decreased proportional to severity of disease. We furthermore demonstrated that fibrinogen concentrate and PCCs, but not factor XIII concentrate, improve clot properties in patient plasma. Whether factor concentrates have clinical and how their use might best be targeted requires additional study.

## ETHICS APPROVAL AND PATIENT CONSENT

The NRES Committee London‐Westminster (12/LO/1417) and the Medical Ethical Committee Hospital Clinic Barcelona (2017/0948) approved the study protocol which was in accordance with the Helsinki Declaration of 1975. All patients gave written informed consent.

## CONFLICTS OF INTEREST

None.

## Supporting information

Table S1‐2

## Data Availability

The data that support the findings of this study are available from the corresponding author upon reasonable request.
