# Supplementary material for: Fibrin clot quality in acutely ill cirrhosis patients: Relation with outcome and improvement with coagulation factor concentrates
Source: Liver Int. 2021 Dec 20;42(2):435–43. doi: 10.1111/liv.15132 (PMC9299765; doi:10.1111/liv.15132)
Supplement: Supplementary file 1 — Table S1‐2 [file LIV-42-435-s001.docx]

**Supplementary table 1**. Endogenous thrombin potential and plasma levels of prothrombin, fibrinogen and factor XIII stratified by low and high SOFA, CLIF-AD, CLIF-ACLF, and Child-Pugh scores and by the presence / absence of liver, renal, coagulation, hemodynamic, respiratory and neurological failure in patients with AD

| ***SOFA (median value 4)*** | | | |
| --- | --- | --- | --- |
|  | Low (≤ 4), n=25 | High (> 4) , n=27 | p |
| ETP, nM IIa * min | 765 (663-896) | 739 (647-924) | ns |
| F II, % | 56 (41-57) | 38 (26-44) | 0,001 |
| Fibrinogen, g/L | 2,6 (1,5-3,0) | 1,5 (1,2-2,5) | 0,02 |
| FXIII, % | 40 (29-72) | 35 (25-45) | ns |
| ***CLIF-AD (median value 60)*** | | | |
|  | Low (≤ 60), n= 26 | High (> 60), n= 26 | p |
| ETP, nM IIa * min | 717 (648-807) | 859 (713-1000) | 0,01 |
| F II, % | 48 (25-63) | 42 (38-55) | ns |
| Fibrinogen, g/L | 2,1 (1,3-2,9) | 2,0 (1,4-3,0) | ns |
| FXIII, % | 39 (29-63) | 34 (26-47) | ns |
| ***Child-Pugh (median value 9)*** | | | |
|  | Low (≤ 9), n=19 | High (> 9 ), n=33 |  |
| ETP, nM IIa * min | 761 (655-954) | 753 (682-892) | ns |
| F II, % | 52 (41-67) | 30 (23-41) | < 0,001 |
| Fibrinogen, g/L | 2,6 (1,6-3,0) | 1,4 (1,1-1,9) | < 0,001 |
| FXIII, % | 35 (25-47) | 37 (29-63) | ns |
| ***Liver failure (Bilirubin >205 µmol/l)*** | | | |
|  | No, n= 47 | Yes, n= 5 | p |
| ETP, nM IIa * min | 750 (660-913) | 804 (692-1017) | ns |
| F II, % | 44 (29-59) | 41 (20-55) | ns |
| Fibrinogen, g/L | 2,0 (1,4-2,9) | 1,8 (0,8-2,7) | ns |
| FXIII, % | 36 (28-56) | 46(32-57) | ns |
| ***Renal failure (Creatinine> 176 µmol/l )*** | | | |
|  | No, n= 52 | Yes, n= 0 | p |
| ETP, nM IIa * min | 763 (669-929) | - | - |
| F II, % | 44 (30-59) | - | - |
| Fibrinogen, g/L | 2,1 (1,4-2,9) | - | - |
| FXIII, % | 36 (28-57) | - | - |
| ***Coagulation failure (INR >2,5)*** | | | |
|  | No, n= 50 | Yes, n= 2 | p |
| ETP, nM IIa * min | 759 (666-918) | 647 | ns |
| F II, % | 44 (30-60) | 14 | 0.002 |
| Fibrinogen, g/L | 2,1 (1,4-2,9) | 0,9 | 0.02 |
| FXIII, % | 36 (28-56) | 38 | ns |
| ***Hemodynamic failure (MAP , 80 mmHg with vasoactive support)*** | | | |
|  | No, n= 51 | Yes, n= 1 | p |
| ETP, nM IIa * min | 758 (633-913) | 675 | ns |
| F II, % | 44 (30-60) | 26 | ns |
| Fibrinogen, g/L | 2,1 (1,4-2,9) | 1,4 | ns |
| FXIII, % | 36 (29-57) | 20 | ns |
| ***Respiratory failure (PaO_2_/FiO_2_ < 200 )*** | | | |
|  | No, n= 52 | Yes, n= 0 | p |
| ETP, nM IIa * min | 755 (664- 908) | - | - |
| F II, % | 39 (25-52) | - | - |
| Fibrinogen, g/L | 2,1 (1,4-2,9) | - | - |
| FXIII, % | 36 (28-57) | - | - |
| **Neurologic failure (Encephalopathy moderate-severe)** | | | |
|  | No, n= 42 | Yes, n= 10 | p |
| ETP, nM IIa * min | 775 (662-945) | 698 (664-762) | ns |
| F II, % | 44 (33-60) | 31 (26-58) | ns |
| Fibrinogen, g/L | 2,2 (1,5-3,0) | 1,5 (1,1-2,7) | ns |
| FXIII, % | 35 (28-56) | 42 (29-58) | ns |
| **Infection** | | | |
|  | No, n= 42 | Yes, n= 10 |  |
| ETP, nM IIa * min | 765 (665-940) | 675(660-726) | ns |
| F II, % | 44 (30-60) | 44 (24-52) | ns |
| Fibrinogen, g/L | 2,1 (1,4-2,9) | 1,5 (1,4-2,9) | ns |
| FXIII, % | 36 (28-56) | 51 (25-57) | ns |

**Supplementary table 2**. Endogenous thrombin potential and plasma levels of prothrombin, fibrinogen and factor XIII stratified by low and high SOFA, CLIF-AD, CLIF-ACLF, and Child-Pugh scores and by the presence / absence of liver, renal, coagulation, hemodynamic, respiratory and neurological failure in patients with ACLF.

| ***SOFA (median value 8)*** | | | |
| --- | --- | --- | --- |
|  | Low (≤ 8), n=24 | High (> 8) , n=33 | p |
| ETP, nM IIa * min | 761 (535-916) | 641 (525-843) | ns |
| F II, % | 38 (27-49) | 23 (17-34) | < 0,002 |
| Fibrinogen, g/L | 2,1 (1,5-2,9) | 1.3 (0,7-2,0) | < 0,009 |
| FXIII, % | 38 (25-55) | 26 (18-34) | < 0,01 |
| ***CLIF-ACLF (median value 87)*** | | | |
|  | Low (≤ 87), n= 28 | High (> 87), n= 29 | p |
| ETP, nM IIa * min | 742 (535-886) | 658 (626-912) | ns |
| F II, % | 37 (24-47) | 26 (19-41) | ns |
| Fibrinogen, g/L | 2,8 (1,2-2,6) | 1,8 (0,9-2,5) | ns |
| FXIII, % | 31 (20-43) | 32 (23-46) | ns |
| ***Child-Pugh (median value 10)*** | | | |
|  | Low (≤ 10), n=26 | High (> 10 ), n=31 |  |
| ETP, nM IIa * min | 638 (510-857) | 759 (603-950) | ns |
| F II, % | 38 (29-50) | 24 (17-36) | 0,001 |
| Fibrinogen, g/L | 2,1 (1,6-2,7) | 1,2 (0,6-2,1) | 0,002 |
| FXIII, % | 32 (23-48) | 29 (21-39) | ns |
| ***Liver failure (Bilirubin >205 µmol/l)*** | | | |
|  | No, n= 32 | Yes, n= 25 | p |
| ETP, nM IIa * min | 849 (627-986) | 626 (509-718) | 0,004 |
| F II, % | 39 (34-50) | 22 (17-31) | < 0,001 |
| Fibrinogen, g/L | 2,4 (1,7-2,7) | 0,8 (0,5-1,8) | < 0,001 |
| FXIII, % | 31 (19-56) | 32 (23-40) | ns |
| ***Renal failure (Creatinine> 176 µmol/l )*** | | | |
|  | No, n= 17 | Yes, n= 40 | p |
| ETP, nM IIa * min | 613 (484-938) | 741 (618-901) | ns |
| F II, % | 33 (20-42) | 34 (20-46) | ns |
| Fibrinogen, g/L | 1,9 (1,4-2,6) | 1,2 (0,5-2,3) | 0,04 |
| FXIII, % | 33 (22-37) | 30 (21-50) | ns |
| ***Coagulation failure (INR >2,5)*** | | | |
|  | No, n= 40 | Yes, n= 17 | p |
| ETP, nM IIa * min | 749 (577-938) | 637 (522-836) | ns |
| F II, % | 38 (31-48) | 20 (16-24) | < 0,001 |
| Fibrinogen, g/L | 2,1 (1,6-2,6) | 0,8 (0,5-1,6) | < 0,001 |
| FXIII, % | 33 (24-48) | 24 (17-37) | ns |
| ***Hemodynamic failure (MAP , 80 mmHg with vasoactive support)*** | | | |
|  | No, n= 37 | Yes, n= 20 | p |
| ETP, nM IIa * min | 738 (585-908) | 639 (516-878) | ns |
| F II, % | 38 (26-47) | 23 (18-35) | 0,008 |
| Fibrinogen, g/L | 2,1 (1,2-2,6) | 1,6 (0,7-2,0) | ns |
| FXIII, % | 37 (23-59) | 27 (19-34) | 0,02 |
| ***Respiratory failure (PaO_2_/FiO_2_ < 200 )*** | | | |
|  | No, n= 46 | Yes, n= 11 | p |
| ETP, nM IIa * min | 736 (548- 896) | 637 (519- 1003) | ns |
| F II, % | 35 (23-46) | 30 (17-41) | ns |
| Fibrinogen, g/L | 1,9 (1,1-2,6) | 1,7 (0,8-2,5) | ns |
| FXIII, % | 32 (22-47) | 29 (18-37) | ns |
| **Neurologic failure (Encephalopathy moderate-severe)** | | | |
|  | No, n= 34 | Yes, n= 23 | p |
| ETP, nM IIa * min | 742 (541-900) | 658 (503-913) | ns |
| F II, % | 35 (20-46) | 28 (20-42) | ns |
| Fibrinogen, g/L | 1,8 (1,3-2,6) | 1,9 (0,8-2,5) | ns |
| FXIII, % | 30 (21-45) | 33 (23-42) | ns |
| **Infection** | | | |
|  | No, n= 33 | Yes, n= 24 |  |
| ETP, nM IIa * min | 641 (519-896) | 736 (571-992) | ns |
| F II, % | 31 (23-45) | 35 (18-43) | ns |
| Fibrinogen, g/L | 1,8 (1,0-2,3) | 1,9 (1,1-2,9) | ns |
| FXIII, % | 29 (23-38) | 39 (18-53) | ns |
